# Strong Shift to ATR-Dependent Regulation of the G_2_-Checkpoint after Exposure to High-LET Radiation

**DOI:** 10.3390/life11060560

**Published:** 2021-06-14

**Authors:** Veronika Mladenova, Emil Mladenov, Michael Scholz, Martin Stuschke, George Iliakis

**Affiliations:** 1 Department of Radiation Therapy, Division of Experimental Radiation Biology, University Hospital Essen, University of Duisburg-Essen, 45122 Essen, Germany; veronika.mladenova@uk-essen.de (V.M.); emil.mladenov@uk-essen.de (E.M.); martin.stuschke@uk-essen.de (M.S.); 2 Institute of Medical Radiation Biology, University Hospital Essen, University of Duisburg-Essen, 45122 Essen, Germany; 3 Biophysics Division, GSI Helmholtzzentrum für Schwerionenforschung GmbH, 64291 Darmstadt, Germany; m.scholz@gsi.de; 4 German Cancer Consortium (DKTK), Partner Site University Hospital Essen, 45122 Essen, Germany; 5 German Cancer Research Center (DKFZ), 69120 Heidelberg, Germany

**Keywords:** ionizing radiation (IR), heavy ions, alpha particles, high-LET radiation, structural chromosomal abnormalities (SCAs)

## Abstract

The utilization of high linear-energy-transfer (LET) ionizing radiation (IR) modalities is rapidly growing worldwide, causing excitement but also raising concerns, because our understanding of their biological effects is incomplete. Charged particles such as protons and heavy ions have increasing potential in cancer therapy, due to their advantageous physical properties over X-rays (photons), but are also present in the space environment, adding to the health risks of space missions. Therapy improvements and the protection of humans during space travel will benefit from a better understanding of the mechanisms underpinning the biological effects of high-LET IR. There is evidence that high-LET IR induces DNA double-strand breaks (DSBs) of increasing complexity, causing enhanced cell killing, owing, at least partly, to the frequent engagement of a low-fidelity DSB-repair pathway: alternative end-joining (alt-EJ), which is known to frequently induce severe structural chromosomal abnormalities (SCAs). Here, we evaluate the radiosensitivity of A549 lung adenocarcinoma cells to X-rays, α-particles and ^56^Fe ions, as well as of HCT116 colorectal cancer cells to X-rays and α-particles. We observe the expected increase in cell killing following high-LET irradiation that correlates with the increased formation of SCAs as detected by mFISH. Furthermore, we report that cells exposed to low doses of α-particles and ^56^Fe ions show an enhanced G_2_-checkpoint response which is mainly regulated by ATR, rather than the coordinated ATM/ATR-dependent regulation observed after exposure to low doses of X-rays. These observations advance our understanding of the mechanisms underpinning high-LET IR effects, and suggest the potential utility for ATR inhibitors in high-LET radiation therapy.

## 1. Introduction

The year 2021 marks the sixtieth anniversary of the first human-operated spaceflight, which ushered in technological and scientific developments that ignited the exploration and exploitation of space [1,2,3]. Activities in science-fiction novels written more than one century ago are now a reality, and will soon become routine. In parallel, the duration of space travel and the number of space missions have increased dramatically. Currently, humans spend more than six months in space on the International Space Station (ISS) [4], and the exploration of Mars with manned missions is a central goal for the coming decades of several national space programs.

There has been significant progress in understanding the effects of space radiation on the homeostasis of living organisms [1,2,3]. Furthermore, the related multiomic, molecular, physiological and behavioral datasets that quickly accumulate provide a valuable roadmap to the analysis of putative health risks associated with spaceflight [5]. Indeed, evaluation of the effects of a 340-day mission onboard the ISS revealed persisting DNA damage, chromosomal inversions, and the shortening of telomeres detectable even 6 months after returning to Earth [5]. These results raise well-founded concerns regarding the risks deriving from the radiation environment in space, and specifically from the biological effects of the energetic high-LET elementary particles and ions it comprises. The radiation environment in low Earth orbits is heterogeneous and consists of galactic cosmic rays (GCRs), solar particle events (SPEs), as well as of electrons and protons trapped (TPs) in the van Allen belts outside the spacecraft. On the other hand, near the Moon or Mars, and in deep space, where a protective magnetic field is almost completely absent, astronauts are exposed to highly energetic heavy ions and solar energetic particles (SEPs) with fluctuating intensities that pose high risks to crew members [4]. Galactic cosmic rays comprise particles of high-LET with increased relative biological effectiveness (RBE), owing to the associated ionization clustering that induces complex DSBs in exposed cells [6,7]. Ionization clusters may also lead to the formation of DSB clusters, comprising two or more DSBs that destabilize chromatin and challenge the DSB repair systems of higher eukaryotes [7,8,9,10].

It has long been known that heavy ions induce a larger proportion of complex structural chromosomal abnormalities (SCAs) than X-rays, from the interaction of more than two DSBs induced in proximity—in one, two or more chromosomes [11,12]. Other studies have revealed that the exposure of human lymphocytes to heavy ions increases the yield of acentric fragments that are often lost during mitosis and form micronuclei that reduce cell viability [13]. Indeed, the identification of a cytogenetic signature that discriminates high- from low-LET IR exposure remains a long-term goal in radiobiology. The advent of fluorescence in situ hybridization (FISH) has revolutionized the analysis of SCAs, and the recent development of sequencing methods that detect genomic alterations paves the way to a bright future in the analysis of IR effects on the genome. However, the random nature of DSB induction in the genomes of irradiated cell populations currently limits the utility of these methodologies. However, single-cell, long-read sequencing promises to overcome these limitations, and powerful methodologies along these lines are being developed and continuously improved [14].

In cells of higher eukaryotes, DSBs are mainly repaired by classical non-homologous end-joining (c-NHEJ) and homologous recombination (HR). However, under certain conditions that are presently under intensive investigation, alternative end-joining (alt-EJ) and single-strand annealing (SSA) also emerge [15,16,17,18,19]. It has previously been shown that the LET-dependent increase in SCAs results from the error-prone processing of DSBs [20]. Notably, with the exception of HR, all DSB repair pathways can catalyze such events, albeit with a different efficiency and cell cycle dependency.

The probability of SCA formation is thought to increase with increasing DSB complexity, because it increases with LET. It is also thought to be higher when DSB clusters form, also because it increases with LET [9,10]. It is widely assumed that complex DSBs and DSB clusters challenge first-line DSB repair pathways such as c-NHEJ and HR, ultimately allowing the engagement of SSA and particularly alt-EJ, which are prone to errors causing SCAs. Indeed, published data suggest that a fraction of resected DSBs in cells exposed to high-LET IR are eventually shunted to SSA or alt-EJ and participate in the formation of SCAs [21,22,23].

The generation of DSBs by low- or high-LET IR initiates a network of cellular responses, resulting in the activation of DNA damage checkpoints. The activation of DNA damage checkpoints in cells exposed to low- or high-LET IR arrests the normal progression of cells at specific points in the cell cycle to facilitate DSB repair [24,25]. Particularly relevant in this regard is the checkpoint activated in the G_2_-phase, because cells in this phase of the cell cycle are competent for all available DSB repair pathways [26,27]. It is commonly thought that this checkpoint is regulated by the ATM kinase with the ATR kinase playing a secondary role. We have recently reported that cells exposed to doses of X-rays below 2 Gy in the G_2_-phase activate a strong G_2_-checkpoint that is epistatically regulated by ATM and ATR, i.e., inhibition of either kinase causes its almost-complete abrogation. This mode of regulation changes at higher X-ray doses, with ATM and ATR now regulating the G_2_-checkpoint both epistatically and independently, i.e., the inhibition of one kinase abrogates the checkpoint only partly and the inhibition of both kinases is required for its complete abrogation [28]. Strikingly, this mode of ATM/ATR regulation is rather specific for cells irradiated in G_2_-phase. Cells irradiated in the S-phase mount when progressing into the G_2_-phase a checkpoint that is exclusively dependent on ATR, and where the inhibition of ATM prolongs rather than suppresses its activation, suggesting that ATM contributes here to checkpoint recovery [29]. However, information about the form of ATM/ATR crosstalk after exposure to high-LET IR is scarce.

Continuing previous studies with X-rays [30,31], and in our effort to better understand the mechanisms underpinning the elevated biological effectiveness of high-LET IR, here we studied the response of two human cell lines—lung carcinoma-derived A549 and colon cancer-derived HCT116 to X-rays, α-particles and ^56^Fe ions, by following the radiosensitivity to killing, the formation of SCAs, as well as activation of the G_2_-checkpoint in cells with suppressed ATM or ATR activity. Our results show, as expected, that A549 and HCT116 cells exhibit increased radiosensitivity to high-LET IR. The reduced cell viability following exposure to α-particles and ^56^Fe ions correlates with an increased incidence of SCAs that may be a manifestation of increased contribution of low-fidelity alt-EJ. We also analyzed the ATM/ATR-dependent regulation of the G_2_-phase checkpoint after exposure to low doses of high-LET IR. In contrast to the epistatic regulation of the G_2_-checkpoint by ATR and ATM following exposure to X-rays [28], and in line with an earlier report [32], we found regulation mainly by ATR after exposure to α-particles and ^56^Fe ions.

## 2. Materials and Methods

### 2.1. Cell Lines and Inhibitors

The human alveolar adenocarcinoma cell line A549, and its ATM-deficient counterpart which is designated here as A459-*ATM*^−^, were maintained in McCoy’s 5A growth medium (Sigma-Aldrich, Darmstadt, Germany), supplemented with 10% newborn calf serum (Capricorn) and 0.5 μg/mL iron (FeSO_4_·7H_2_O), at 37 °C, in an atmosphere of 5% CO_2_ in air. HCT116 colon cancer cells were grown in McCoy’s 5A growth medium (Sigma-Aldrich), supplemented with 10% fetal calf serum (Sigma-Aldrich, Darmstadt, Germany). Exponentially growing cells were subcultured every second day to achieve maximum confluence of about 75%.

CRISPR/Cas9 technology was utilized for the generation of A549-*ATM*^−^ cells. For this purpose, A549 cells were transfected with Cas9 expression plasmids, co-expressing GFP, together with a plasmid constructed to express gRNA (5′-ATCATTAAGTACTAGACTCA-3′), targeting exon 2 of the *ATM* gene. Cell sorting based on GFP expression was utilized to select individual GFP-positive clones, in which the activity of ATM was monitored by immunofluorescence detecting pATM-S1981, a marker of ATM activation; negative clones were selected and expanded for further characterization.

All inhibitors were dissolved in dimethyl sulfoxide (DMSO) and were added to the culture medium one hour before irradiation. The ATR inhibitor, VE-821 (Haoyuan ChemExpress, Shanghai, China; CAS. no: 1232410-49-9), referred to as ATRi, was applied at a concentration of 5 μM, whereas the ATM inhibitor KU55933 (ATMi), (Haoyuan ChemExpress, Shanghai, China; CAS. no: 587871-26-9) was applied at a concentration of 10 μM. In experiments measuring cell survival by colony formation as endpoints, cells were transferred to inhibitor-free growth medium 24 h after irradiation.

### 2.2. Radiation Exposure

Irradiations with low-LET X-rays (effective photon energy ~70–90 keV, LET ~ 1–2 keV/µm) were carried out in Essen using an X-ray machine (“Isovolt 320HS”, Seifert/Pantak, General Electric-Pantak, Frankfurt am Main, Germany). Tube voltage and current were set to 320 kV and 10 mA, respectively, and a 1.65 mm aluminum filter (GE-Healthcare, Frankfurt am Main, Germany) was used to absorb “soft” X-rays. The dose rate was ~3.4 Gy/min and was determined using an in-field ionization monitor calibrated with a PTB dosimeter (Physikalisch-Technische Bundesanstalt, Braunschweig, Germany). The radiation dose was confirmed with Fricke’s chemical dosimetry. An even dose distribution within the irradiation field was achieved by rotating the radiation table. Cells were irradiated at a distance of 500 mm. Immediately after irradiation, cells were returned to the incubator.

Exposure to heavy ions (HIs) was carried out at the GSI (Helmholtzzentrum für Schwerionenforschung GmbH) in Darmstadt, Germany. Typically, cells were seeded in 25 cm^2^ tissue culture flasks and were incubated for at least 24 h at 37 °C in Essen. The following day, cells were transported in an insulated container filled with warm pads and allowing active heating to maintain the temperature of the cells close to 37 °C. Upon arrival at the GSI, cells were promptly incubated at 37 °C under standard growth conditions, and when possible, were allowed to recover for several hours from the transportation stress. Cells were exposed to 1 GeV/amu ^56^Fe ions, LET = 150 keV/μm. Dosimetry was carried out with a calibrated farmer chamber (PTW, Freiburg, Germany). The absolute particle fluence was measured with a calibrated ionization chamber (GSI, Darmstadt, Germany) at the beam exit window, and the homogeneity of the scanned field was regularly checked using radiochromic EBT films (Gafchromic, Ashland, OR, USA). LET was calculated using “ATIMA”, a program developed at the GSI. After radiation exposure, cells were immediately transported to Essen, while maintaining them close to 37 °C. When necessary, during transportation, samples were transferred to ice to measure the kinetics of responses under investigation. The limited availability of HI for biological experiments compromised the number of repeats that could be performed.

The device for α-particle irradiation has previously been described in detail [33], and consists of two cylindrical chambers, one containing the cell target (target chamber) and the other accommodating the source and the collimator (source chamber). The ^241^Am α-particle source was plated as an Am_2_O_3_ compound (1.2 × 10^8^ Bq, diameter 55 mm, collimator 10 mm) into a silver foil and was covered with a gold layer of 2 μm in thickness. An aluminum ring with inner diameter of 50 mm was used to delimit the source active area. For α-particle exposures, sterile glass rings (inner diameter of 45 mm and height of 22 mm) with a 1.5 μm thick Mylar foil bottom were used as previously described [33]. Cells were grown for 24 h on Mylar foil and irradiated with 5.49 MeV (3.4 MeV at the cell surface) ^4^He ions generated from the ^241^Am source. During the irradiation, the source chamber was flushed with helium while the target chamber contained normal air. A homogenous dose distribution was achieved by rotating the α-particle source and by wobbling the collimator. The dose rate was 1.32 Gy/min, and the mean LET was 124 keV/μm [33]. Non-irradiated cells were exposed to the same environmental conditions (temperature fluctuations and low CO_2_ concentration) as cells subjected to radiation.

### 2.3. Colony Formation Assay

To assess the colony-forming ability of A549, HCT116 and A459-*ATM*^−^ cells following the exposure to IR of different LET, cells were plated in triplicate four hours after exposure to X-rays and α-particles, or immediately upon arrival in Essen, which was typically four to five hours after irradiation with ^56^Fe ions. Cells were grown for 11 days and stained with 1% crystal violet dissolved in 70% ethanol. Colonies were counted using a low-magnification binocular microscope. Alternatively, dishes were scanned, and the colonies were scored on the digitized images.

### 2.4. Multicolor Fluorescence In Situ Hybridization (mFISH)

To score SCAs, mFISH analysis was employed 48 h after irradiation. To accumulate cells at metaphase, colcemid (Biochrom AG, Berlin, Germany) was added over 2–3 h at a concentration of 0.1 μg/mL. Cells were harvested by trypsinization and incubated for 15 min in 10 mL hypotonic solution (75 mM, KCl). Subsequently, cells were fixed in 10 mL ice-cold fixative—3:1 methanol (Sigma-Aldrich, Darmstadt, Germany):acetic acid (Carl Roth GmbH & Co., Karlsruhe, Germany) and kept at 4 °C overnight. After several washes with the fixative, metaphase spreads were prepared and dried for 24 h at room temperature. mFISH was performed using a 24 × Cyte Multicolor FISH probe for human chromosomes (MetaSystems, Atlussheim, Germany), according to the manufacturer’s protocol. An automated imaging system (MetaSystems, Atlussheim, Germany) was used to obtain high-quality images of metaphase chromosomes. For the metaphase search, the M-Search module of the Metafer software using the 10× air objective of a Zeiss microscope (AxioImager.Z2, Zeiss, Jena, Germany) was used. Metaphases were captured at a magnification of 63×, using the AutoCapt setting of the Metafer software. Images were analyzed using Isis Software. For analysis, at least 50 metaphases were scored in each of three independent repeats of each experiment—except for ^56^Fe ions irradiation, where only one experiment could be carried out.

### 2.5. Flow Cytometry Analysis of Mitotic Index Using H3-pS10 Staining

Two-parameter flow cytometry was employed to simultaneously measure DNA content by propidium iodide (PI) staining and mitotic cells by quantification of the phosphorylated histone H3 at Serine 10 (H3-pS10). Briefly, 0.6–1.0 × 10^6^ cells were fixed in 70% ice-cold ethanol and were permeabilized for 15 min in ice-cold PBS supplemented with 0.25% Triton X-100. Cell pellets were incubated in 0.05% Tween-20, 1% BSA in PBS for 45 min at RT, followed by incubation with primary rabbit anti-H3-pS10 specific antibody (Abcam PLC, Cambridge, United Kingdom) for 2 h at RT. Cells were washed three times with PBS and incubated in AlexaFluor 488-conjugated goat-anti rabbit-IgG (Thermo Fisher Scientific, Waltham, USA). Finally, DNA was stained with PI for 30 min at 37 °C. All incubation steps were performed under gentle agitation. Analysis was carried out in a Gallios flow cytometer (Beckman Coulter, Krefeld, Germany) by measuring 2 × 10^4^ cells per sample; proper gating was applied to select H3-pS10-positive events that represented mitotic cells. The mitotic index (MI) was determined as the fraction of cells in mitosis and is shown normalized to the MI of non-irradiated controls. The actual MIs of the controls that were used for normalization are given in the legends of the corresponding figures.

### 2.6. Indirect Immunofluorescence for Detection of pATM-S1981 Foci

Auto-phosphorylation of ATM at S1981 is a marker for ATM activation. Therefore, to detect ATM deficiency in selected A549 clones after CRISPR/Cas9 treatment, indirect immunofluorescence was applied to visualize pATM-S1981 foci in cells exposed to X-rays, as described elsewhere [28]. Briefly, cells were irradiated with 1 Gy, and 1 h later were fixed in fixation solution (3% PFA, 2% sucrose in 1 × PBS) for 15 min at room temperature. Cells were permeabilized with P-solution (0.5% Triton X-100, 50 mM EDTA pH 8.0, and 50 mM Tris-HCl pH 7.4) for 10 min. After permeabilization, the cells were washed once with PBS and were blocked overnight in PBG blocking buffer (0.2% gelatin, 0.5% BSA fraction V in 1 × PBS). The primary antibody (mouse monoclonal anti-ATM-S1981, clone 10H11.E12) was diluted 1:400 in PBG and the samples were incubated for 2 h at room temperature. After 3 consecutive washes with PBS, samples were incubated for 1.5 h with the corresponding AlexaFluor 488-conjugated secondary antibody, diluted 1:400 in PBG. After two washes with PBS, samples were counterstained with 0.2 μg/mL DAPI solution, washed with PBS, and mounted in antifade mounting media. Foci were detected under a Leica TCS SP5 confocal microscope (Leica Microsystems, Wetzlar, Germany).

## 3. Results

### 3.1. Increased Radiosensitivity after Exposure to High-LET IR

**Figure 1:**
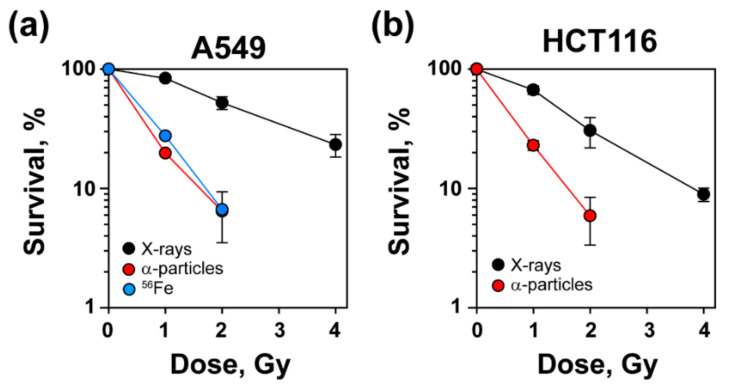
Increased radiosensitivity after exposure to high-LET IR. (**a**) A549 cells were exposed to X-rays (70–90 keV, LET = 1–2 keV/μm), ^241^Am α-particles (3.4 MeV at cell surface, LET = 124 keV/μm), and ^56^Fe ions (1 GeV, LET = 150 keV/μm) and plated for colony formation 4 h later. Plating efficiency (PE) (50.3–56.5%). (**b**) HCT116 cells exposed to X-rays and α-particles under similar conditions. PE (59.2–73.6%). Shown are the mean ± SD from three experiments—except for ^56^Fe irradiation, which reflects one experiment.

The obtained results of A549 and HCT116 cells exposed to increasing doses of low- or high-LET IR and plated for colony formation 4 h later are summarized in Figure 1 and Supplementary Table S1. As expected, A549 cells exposed to high-LET α-particles or ^56^Fe ions exhibited increased radiosensitivity compared to X-rays (Figure 1a). Increased radiosensitivity after exposure to high-LET IR was also documented in HCT116 cells exposed to α-particles (Figure 1b).

### 3.2. High-LET IR Increases the Incidence of SCAs

**Figure 2:**
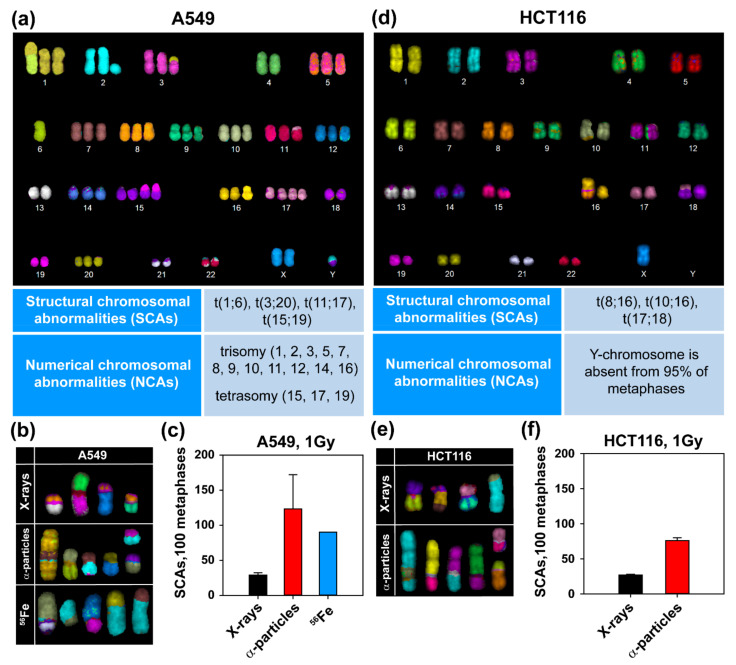
Increased formation of SCAs after exposure to high-LET IR. (**a**) mFISH karyotype map of A549 cells with annotation of constitutive SCAs and NCAs. (**b**) Representative SCAs in A549 cells, exposed to different forms of IR. (**c**) Quantitative analysis of SCAs in A549 cells exposed to X-rays, α-particles and ^56^Fe ions. (**d**) As in (**a**), but for HCT116 cells. (**e**) As in (**b**), but for HCT116 cells. (**f**) As in (**c**), but for HCT116 cells. At least 50 metaphases were scored for each condition. Data represent the mean ± SD from three experiments. Results for ^56^Fe ions are from a single experiment.

We next assessed SCA formation in A549 and HCT116 cells after exposure to high- and low-LET IR. For better resolution in the detection of genomic alterations, we employed mFISH analysis. As a first step, we generated a karyotype map of A549 and HCT116 cells (Figure 2a,d, upper panels). This analysis revealed trisomy and even tetrasomy in multiple chromosomes, as well as constitutive translocations in A549 cells that are summarized in Figure 2a, lower panel. On the other hand, HCT116 cells had a nearly normal karyotype with a modal chromosome number of 45 and three constitutive SCAs; only Y-chromosome was absent from 95% of the metaphases (Figure 2d). Exposures to 1 Gy of X-rays, α-particles or ^56^Fe ions induced multiple SCAs in A549 cells (see Figure 2b for examples). The incidence of SCAs was low after exposure to X-rays (Figure 2c), but increased significantly after exposure to ^56^Fe ions or α-particles (Figure 2c). Similar results were obtained in HCT116 cells exposed to X-rays or α-particles (Figure 2e,f). The increased incidence of SCAs with increasing LET correlates well with the documented increase in cell lethality observed in Figure 1, both for A549 and HCT116 cells.

### 3.3. High-LET IR Alters the ATM/ATR Dependent Regulation of the G_2_-Checkpoint

**Figure 3:**
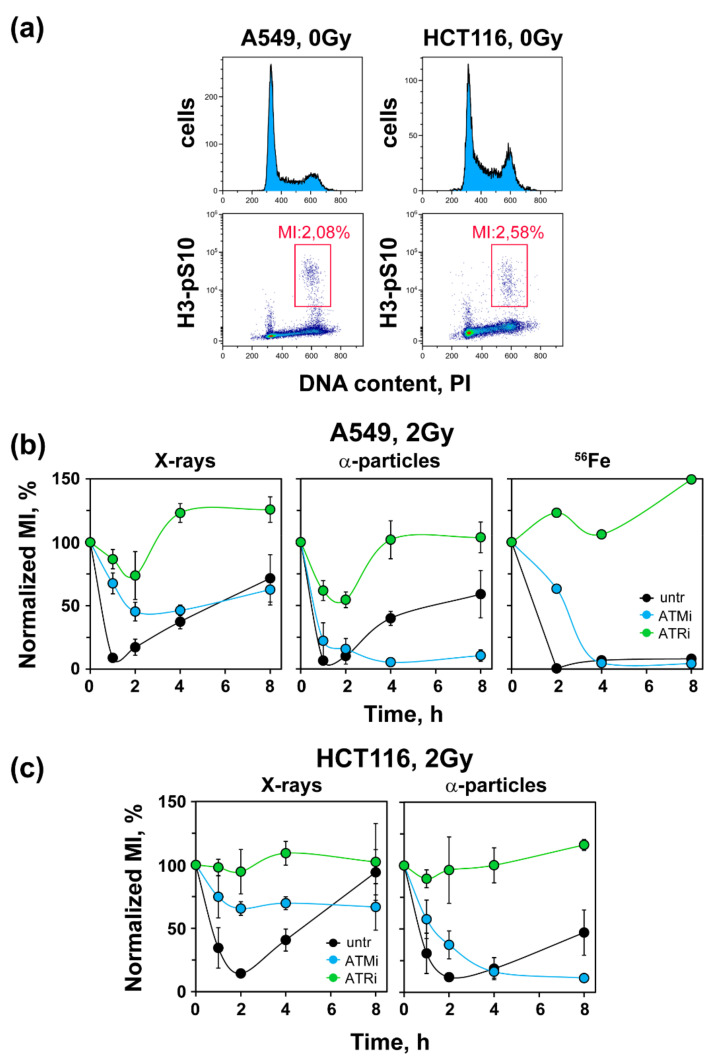
Uncoupling of the ATM/ATR functional module after exposure to low doses of high-LET IR. (**a**) Cell cycle distribution and dot plots of A549 and HCT116 cells stained with PI and H3-pS10 to determine the MI. Shown are the gates used. (**b**) Normalized MI as a function of time in A549 cells exposed to 2 Gy of X-rays, α-particles or ^56^Fe ions. ATMi (10 μM) or ATRi (5 μM) were administered 1 h before irradiation and kept during the course of incubation before collection and analysis. The raw MIs were between 1.57 and 2.26, 1.48 and 2.21, and 2.28 and 2.35 for exposures to X-rays, α-particles and ^56^Fe, respectively. (**c**) As in Figure 3b, but for HCT116 cells. The raw MIs were between 1.84 and 3.13, and 1.72 and 2.95, for exposures to X-rays and α-particles, respectively. All experiments, except those with ^56^Fe which reflect one experiment, were performed in triplicate. Data represent the mean ± SD.

We also inquired how low doses of high-LET IR affect the regulatory organization of the G_2_-checkpoint. To study the G_2_-checkpoint regulation specifically in G_2_-phase irradiated cells, we applied two-parameter flow cytometry as outlined in the Materials and Methods or described earlier [28] (Figure 3a).

When G_2_-phase cells are irradiated in the G_2_-phase, they activate the G_2_-checkpoint, which delays their progression into mitosis and manifests as a decrease in MI. This reduction reflects during the first 4–6 h post-irradiation response of cells irradiated in the G_2_-phase. A549 cells exposed to 2 Gy of X-rays activated a strong checkpoint, as expected, that caused a reduction in the MI to nearly zero at 1–2 h, recovering at later times (Figure 3b, left panel). Treatment with ATMi partly suppressed this checkpoint response, whereas treatment with ATRi abrogated the checkpoint almost completely. These results confirm our previous observations of ATM and ATR involvement in checkpoint activation after the exposure of A549 cells to low doses of X-rays [28], but point to a dominance of ATR under the conditions used in the present set of experiments.

Exposure of A549 cells to 2 Gy of α-particles caused a similarly strong activation of the G_2_-checkpoint, which also recovered to about 50% at 8 h (Figure 3b, middle panel). Notably, however, after exposure to this high-LET radiation modality, ATMi prolonged the checkpoint, suggesting a mechanistic shift in its function with ATM now contributing to checkpoint recovery but not to checkpoint activation. On the other hand, ATRi still conferred a strong, albeit incomplete, suppression of the G_2_-checkpoint, suggesting that ATR remains central in the activation of the G_2_-checkpoint after exposure to high-LET IR. A qualitatively similar response was also observed when A549 cells were exposed to 2 Gy of ^56^Fe ions (Figure 3b, right panel). Here, again, a strong G_2_-checkoint was activated at 2 h that persisted up to 8 h. This checkpoint remained unaffected by ATMi, but was completely abrogated after treatment with ATRi.

The exclusive role of ATM in the recovery, but not activation, of the G_2_-checkpoint after exposure to α-particles was also confirmed in HCT116 cells. Exposure of HCT116 cells to 2 Gy of X-rays resulted in a precipitous drop of MI at 1–2 h post-IR, which started recovering at 4 h and reached pre-irradiation levels at 8 h (Figure 3c). Here, again, ATMi partly and ATRi completely abrogated G_2_-checkpoint activation (Figure 3c, left panel). Exposure of HCT116 cells to α-particles caused a decrease in MI similar to X-rays, but the recovery at 8 h was incomplete. Here, again, ATMi prolonged while ATRi fully abrogated the G_2_-checkpoint. These findings confirm a pivotal role for ATR in the activation of the G_2_-checkpoint following exposure to high-LET IR. It is worth mentioning that ATM exerted a similar checkpoint-prolonging function in cells exposed to X-rays in the S-phase [29].

**Figure 4:**
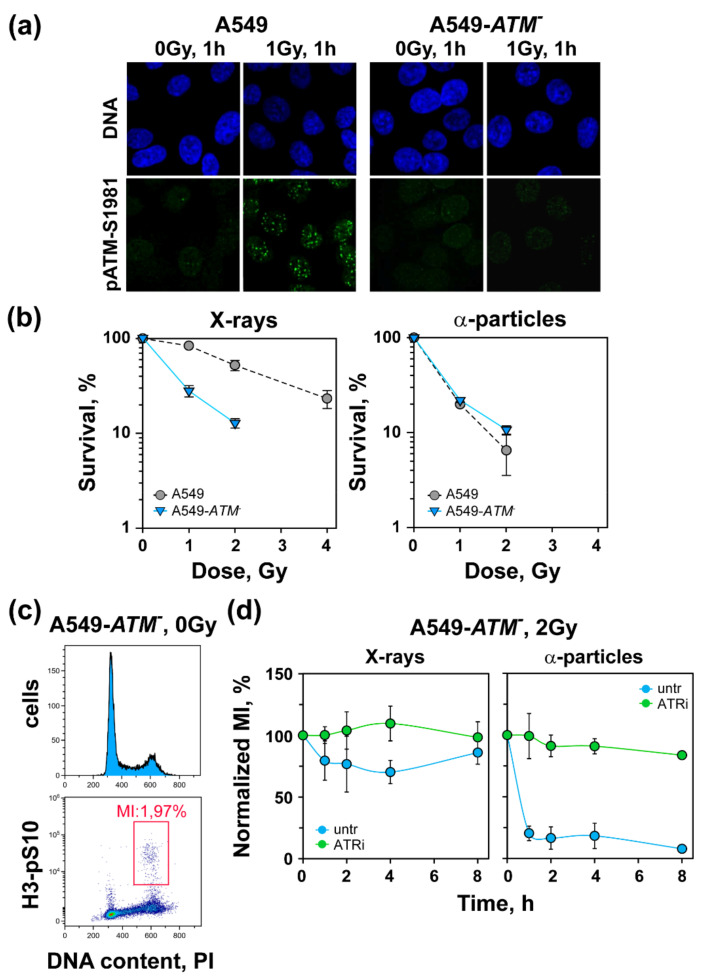
Uncoupling of the ATM/ATR functional module after exposure to low doses of high-LET IR. (**a**) Representative images of pATM-S1981 foci in A549 and A549-*ATM*^−^ cells. (**b**) Survival of A549-*ATM*^−^ cells exposed to X-rays or α-particles, PE (19.8–24.7%). Data show the mean ± SD from three experiments. (**c**) Cell cycle distribution and dot plots of ATM deficient cells stained with PI and H3-pS10 to determine the MI. Shown is the gate used in MI analysis. (**d**) Normalized MI of A549-*ATM*^−^ cells exposed to 2 Gy of X-rays or α-particles. Raw MIs were between 2.12 and 2.73 and 1.53 and 2.95 for cells exposed to X-rays and α-particles, respectively. Data represent the mean ± SD from three experiments.

To confirm the role of ATM in the recovery rather than the activation of the G_2_-checkpoint after exposure to high-LET IR, we generated an ATM-deficient cell line, A549-*ATM*^−^, as outlined in the Materials and Methods. Figure 4a shows the IF results of pATM-S1981 accumulation at DSBs in the selected clone, as well as in parental A549 cells. It is evident that while parental cells exposed to 1 Gy X-rays and analyzed 1 h later developed robust pATM-S1981 foci, the selected clone failed to do so. We conclude that this clone was ATM-deficient. In line with such ATM deficiency, the clone was also highly radiosensitive to X-ray-induced killing (Figure 4b). Notably, genetic inactivation of ATM generated stronger radiosensitization than treatment with ATMi (Supplementary Table S1), suggesting that at the concentration employed, ATM inhibition was incomplete. Interestingly, ATM-deficient cells exposed to α-particles failed to show increased cell killing over X-rays (Figure 4b, right panel).

We next tested the G_2_-checkpoint in A549-*ATM*^−^ cells (Figure 4c,d). It is evident that after exposure to X-rays, A549-*ATM*^−^ cells showed defective activation of the G_2_-checkpoint in line with the role of ATM in its full activation under these conditions. Here, again, ATRi treatment completely abrogated residual checkpoint activation (Figure 4d, left panel). Notably, exposure of ATM deficient cells to α-particles resulted in a precipitous drop of MI, detectable at 1 h after IR, which failed to recover up to 8 h post-irradiation. This strong G_2_-checkpoint was fully ATR-dependent, because the administration of ATRi completely abrogated its activation (Figure 4d, right panel). Collectively, these results substantiate the dominant role of ATR in checkpoint activation after low doses of low-LET IR and demonstrate its exclusive role in this function after exposure to high-LET IR. In contrast to this preservation of function for ATR with changing LET, ATM loses its role in the activation of the G_2_-checkpoint after exposure to high-LET IR and contributes only to its recovery, as it also does after the exposure of S-phase cells to low-LET IR.

### 3.4. Effects of ATM and ATR Inhibition on Cell Survival and SCA Formation in A549 Cells

**Figure 5:**
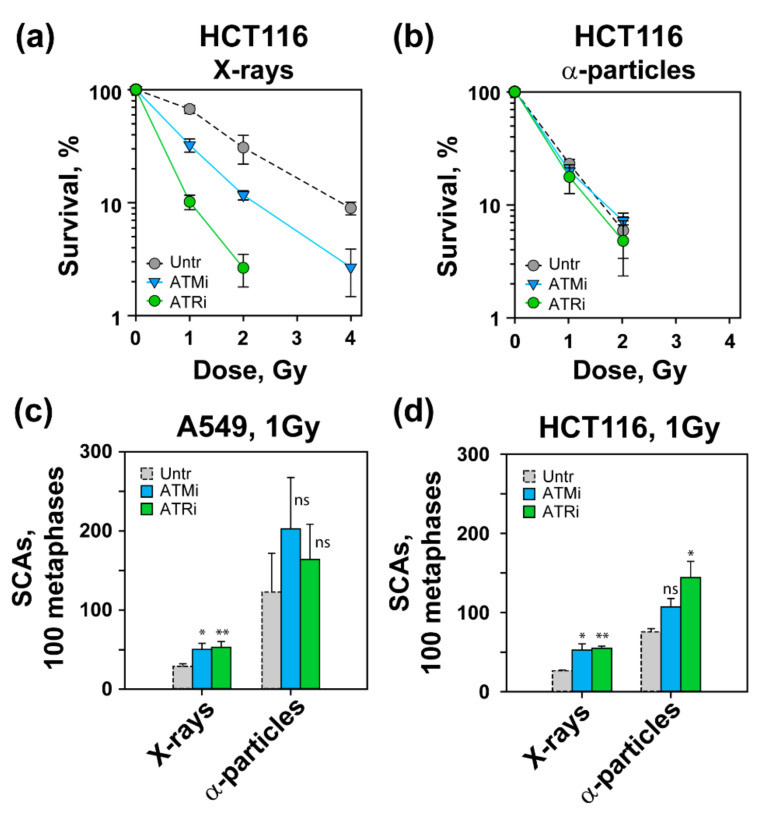
Effect of ATM and ATR inhibitors on cell viability and SCA formation. (**a**,**b**) Colony formation assay of HCT116 cells exposed to different IR modalities. PE_ATMi_ (56.9–57.1%), PE_ATRi_ (34.8–35.7%). (**c**,**d**) SCAs in A549 and HCT116 cells treated with ATMi or ATRi for 24 h. Cells were collected 48 h post-irradiation for SCA scoring. For A549 cells * (*p* = 0.0116), ** (*p* = 0.0067), ns (*p* = 0.1645, *p* = 0.3434), for HCT116 cells * (*p* = 0.0449 and 0.0435), ** (*p* = 0.0044), ns (*p* = 0.0599). Data represent the mean ± SD from three experiments.

To extend the above observations, we studied the effect of ATM and ATR inhibition on cell survival and SCA formation. Our results show that treatment with ATMi or ATRi radiosensitized A549 cells exposed to low-LET, X-ray, but not measurably to those exposed to α-particles or ^56^Fe ions (Supplementary Table S1). On the other hand, when HCT116 cells exposed to X-rays were treated with ATMi, a significant increase in radiosensitization was documented (Figure 5a). In addition, an interesting phenomenon was observed in HCT116 cells exposed to X-rays in the presence of ATRi. Under these conditions, ATRi treatment rendered HCT116 cells extremely radiosensitive, which may reflect their genetic background (Figure 5a) (see Discussion). However, similar to A549 cells, treatment with ATMi or ATRi failed to radiosensitize HCT116 cells exposed to α-particles (Figure 5b).

To compare the effect of ATMi and ATRi at the cell survival level with their impact on SCA formation, we carried out chromosome analysis as described above (Figure 5c,d). Figure 5c shows that in A549 cells exposed to 1 Gy of X-rays, ATMi or ATRi caused statistically significant increases in SCAs (~50%). After exposure to α-particles, ATMi, but not ATRi, increased the incidence of SCAs, but the effect failed to reach statistical significance (Figure 5c). We also examined the effect of ATM and ATR inhibition on SCA formation in cells exposed to ^56^Fe ions, which followed the trends of the cells exposed to high-LET α-particles (data not shown). Moreover, we also studied the impact of ATMi and ATRi on SCA formation in HCT116 cells after exposure to X-rays or α-particles. Figure 5d shows a similar number of SCAs in inhibited HCT116 cells as shown for A549 after exposure to X-rays. However, ATMi slightly increased SCA formation in α-particle-irradiated HCT116 cells, but the effect failed to reach statistical significance. On the other hand, ATRi increased SCAs in α-particle-irradiated HCT116 cells.

## 4. Discussion

### 4.1. High Incidence of SCAs and Increased Cell Killing after Exposure of Cells to High-LET-IR

It is thought that the exposure of cells to high-LET IR increases cell killing over that generated by low-LET IR by altering the rules of DSB repair pathway engagement [7,9,34]. There are extensive investigations and speculations regarding the mechanisms underpinning such changes. Some studies consider error-free HR as a major contributor to DSB repair after exposure to high-LET IR [35], whereas others emphasize the role of c-NHEJ [36]. However, it is well-documented that in addition to c-NHEJ and HR, alt-EJ also engages to process DSBs, and that this engagement increases with increasing LET [9,31,34,37].

Our results show an LET-dependent increase in SCAs in both A549 and HCT116 cells, which are considered hallmarks for alt-EJ engagement and are unlikely to be generated by HR. SCAs can be lethal or carcinogenic events, and therefore the engagement of repair pathways favoring their formation, such as alt-EJ, must be carefully balanced by the cell. A clear net benefit must arise for genomic stability to justify the recruitment of alt-EJ. We assume therefore that following exposure to high-LET IR, forms of DSBs are generated that engage first-line DSB repair pathways such as HR or c-NHEJ. However, when these repair pathways are challenged and occasionally ultimately fail owing to the complexity of the DSB, alt-EJ becomes the last resort—a form of backup. The choice for the cell is therefore to either utilize alt-EJ despite the associated risks, or to leave the DSB unprocessed. It is likely that the degree of genomic preservation ensured by alt-EJ engagement is higher than that associated with the option of leaving the DSB unprocessed and risk the loss of genomic material. Notably, such decisions are likely required for only a small fraction of DSBs that are actually ultimately shunted to alt-EJ. In addition, the majority of alt-EJ events will rejoin the correct ends and will not lead to SCAs. Thus, infrequent utilization, as a last resort scenario, may underpin the evolutionary appearance and preservation of alt-EJ.

### 4.2. Altered Regulation of the G_2_-Checkpoint after Exposure to High-LET IR

We have recently demonstrated intriguing contributions and crosstalk between ATM and ATR in the regulation of the G_2_-checkpoint in cells exposed to low doses of low-LET IR in the G_2_-phase. Indeed, we were able to demonstrate that under these conditions, ATM and ATR operate in the form of a functional module within which the two kinases epistatically regulate G_2_-checkpoint activation [28,29]. Our results with high-LET IR show a strong shift in the regulatory organization of the checkpoint which is now almost exclusively regulated by ATR. Indeed, the contribution of ATM operates in the opposite direction, functioning as a checkpoint-recovery modulator. Importantly, this role can be confirmed either by using inhibitors or genetic inactivation of the ATM kinase. We conclude that following exposure to high-LET IR, the function of the ATM/ATR module changes profoundly from the function detected after exposure to low-LET IR [28,29]. The detailed mechanistic analysis of this modification will require further investigations.

A similar dominant role of ATR in the G_2_-checkpoint has been reported before and after the exposure of cells to carbon ions [32], and our results with α-particles and ^56^Fe ions are in general agreement with the findings of that report. Another report showed that ATR inhibition abrogates the G_2_-checkpoint, induces micronuclei, and causes cell radiosensitization after exposure to carbon ions [38]: all findings in line with our observations.

The increased functional involvement of ATR in the G_2_-checkpoint after high-LET IR suggests the engagement of DSB repair pathways utilizing DNA-end resection and therefore generating the ssDNA required to activate ATR. Alt-EJ falls in this category of DSB repair pathways. DNA-end resection exposes microhomologies and larger regions of homology that support alt-EJ, and, if long enough, SSA as well [39]. SSA is highly error-prone due to the obligate deletion of the intervening sequence between DNA repeats, and may also lead to SCA formation [40]. Further studies are required to decipher the exact contribution of resection-dependent mechanisms to the processing of high-LET-induced complex DSBs and DSB-clusters and to confirm or rule out the proposed engagement of error-free HR.

### 4.3. LET-Dependent Radiosensitization and SCA Induction by ATMi or ATRi

HCT116 cells are more sensitive to X-rays than A549 cells. This may reflect the documented *MLH1* or *MSH2* deficiency of HCT116 cells [41], as well as the observation that HCT116 cells express low levels of MRE11, a major component of the MRN complex involved in DNA end resection [42,43]. Our study also addresses the impact of ATM and ATR inhibition on the radiosensitivity of A549 and HCT116 cells. Indeed, treatment of cells with ATMi or ATRi radiosensitizes cells only to low-LET IR (Figure 4). This effect correlates with the effects of the inhibitors on SCA formation, where more significant relative increases are observed for cells exposed to low-LET IR.

Our observations are also in line with previous studies showing that the transient inhibition of ATM causes the accumulation of persistent chromosome aberrations [44,45]. Inhibition of ATM suppresses the formation of IR-induced sister chromatid exchanges (SCEs), a process attributed to homologous recombination-mediated repair [46]. ATM deficiency also causes 11q23 chromosome translocations—the most frequent chromosome abnormality in secondary leukemia [44]. It is likely that the decreased fidelity of DSB repair by the inappropriate regulation of HR that leads to the engagement of alt-EJ in ATM-inhibited cells increases the incidence of SCAs after exposure to low-LET IR.

Recently, ATM and ATR have been selected as potential pharmacological targets in the CONCORDE platform—the first phase Ib randomized, open-label, multi-institution, multi-arm clinical trial seeking to determine the safety profile of several DDR inhibitory agents in combination with fixed-dose radical radiotherapy in locally advanced non-small cell lung carcinomas (LA-NSCLC). Non-malignant cells possess a functional G_1_-checkpoint, in contrast to most NSCLC cells, it is proposed that selective tumor radiosensitization can be achieved by abrogation of the G_2_-checkpoint using inhibitors of ATM or ATR [47]. The altered regulation of the G_2_-checkpoint documented in our study, as well as in previous studies, after exposure to high-LET IR should be helpful in the goals of this platform, when high-LET IR modalities become integrated as treatment options.

### 4.4. Concluding Remarks

The present study has confirmed the enhanced cellular radiosensitivity following exposure to high-LET IR and reinforces its correlation with the increased incidence of SCAs that, in turn, implies the increased engagement of error-prone DSB repair pathways. The prevailing role of ATR in the regulation of G_2_-checkpoint points to mechanistic shifts in the crosstalk between ATM and ATR in the regulation of this endpoint and emphasizes the increased role of DNA end-resection in DSB repair and the potential of ATR inhibitors in the clinical setting. Collectively, this information is relevant to the utilization of high-LET IR in cancer therapy and to radiation protection during space travel.

## Data Availability

Not applicable.

## Supplementary Materials

The following are available online at https://www.mdpi.com/article/10.3390/life11060560/s1. Table S1: Survival, (%) of A549, HCT116 and A549-ATM- cells exposed to different radiation modalities in the presence or not of ATMi or ATRi.
