# Supplementary material for: Strong Shift to ATR-Dependent Regulation of the G2-Checkpoint after Exposure to High-LET Radiation
Source: Life (Basel). 2021 Jun 14;11(6):560. doi: 10.3390/life11060560 (PMC8232161; doi:10.3390/life11060560)
Supplement: Supplementary file 1 [file life-11-00560-s001.zip › life-1241129-SI.pdf]

Supplementary Table S1

	A549, X-rays		
untr	Dose	Average	StDev
	0	100	0
	1	83.80571	2.144201
	2	52.33372	6.466724
	4	23.29663	4.976602

	A549, α-par cles		
untr	Dose	Average	StDev
	0	100	0
	1	19.7628	0.322853
	2	6.449479	2.936311
	4		

	A549, 56Fe		
untr	Dose	Average	StDev
	0	100	0
	1	27.56909	0
	2	6.665007	0
	4		

	HCT116, X-rays		
untr	Dose	Average	StDev
	0	100	0
	1	66.99323	5.559288
	2	30.59344	8.715656
	4	8.924652	1.152503

	HCT116, α-par cles		
untr	Dose	Average	StDev
	0	100	0
	1	22.97645	2.167690
	2	5.893734	2.545548
	4		

	A549, X-rays		
ATMi	Dose	Average	StDev
	0	100	0
	1	57.2896	3.455962
	2	31.24313	7.74396
	4	12.12666	0.14793

	A549, α-par cles		
ATMi	Dose	Average	StDev
	0	100	0
	1	18.05698	3.705604
	2	4.94844	2.293736
	4		

	A549, 56Fe		
ATMi	Dose	Average	StDev
	0	100	0
	1	34.27656	0
	2	5.051754	0
	4		

	HCT116, X-rays		
ATMi	Dose	Average	StDev
	0	100	0
	1	32.23651	4.340011
	2	11.64852	1.104801
	4	2.669719	1.200524

	HCT116, α-par cles		
ATMi	Dose	Average	StDev
	0	100	0
	1	19.74808	1.418262
	2	7.203752	0.573682
	4		

	A549, X-rays		
ATRi	Dose	Average	StDev
	0	100	0
	1	58.42846	10.23177
	2	27.64809	2.732089
	4	13.77974	2.303963

	A549, α-par cles		
ATRi	Dose	Average	StDev
	0	100	0
	1	21.02131	5.562599
	2	5.917196	2.763893
	4		

	A549, 56Fe		
ATRi	Dose	Average	StDev
	0	100	0
	1	23.15604	0
	2	5.35318	0
	4		

	HCT116, X-rays		
ATRi	Dose	Average	StDev
	0	100	0
	1	10.14128	1.496318
	2	2.633585	0.845743
	4		

	HCT116, α-par cles		
ATRi	Dose	Average	StDev
	0	100	0
	1	17.58472	5.039241
	2	4.773974	2.441065
	4		

	A549-*ATM*-, X-rays		
untr	Dose	Average	StDev
	0	100	0
	1	27.7046	3.765331
	2	12.67171	1.44016
	4		

	A549-*ATM*-, α-par cles		
untr	Dose	Average	StDev
	0	100	0
	1	21.84895	1.226778
	2	10.65420	1.064711
	4		

Supplementary Table S1. *Survival, (%) of A549, HCT116 and A549-ATM^{-} cells exposed to different radiation modalities in the presence or not of ATMi or ATRi.*

Data represent averages and standard devia ons from three independent experiments. Data for A549 cells, exposed to ^{56}Fe is generated from a single experiment. Values included in the Supplementary Table S1 were used to plot the graphs for survival experiments, presented in Figure 1, Figure 4b and Figures 5a, b.
